# St. Bartholomew’s Hospital Reports

**Published:** 1886-10

**Authors:** 


ST. BARTHOLOMEW'S HOSPITAL REPORTS. EDITED BY W. H. CHURCH, M. D., AND JOHN LANGTON, F. R. C. S. *Vol. XXI. London:* SMITH, ELDER & CO., *1885.*

The regular articles of the reports are preceded by a List of Subscribers, a Memorial Article on Francis Harris, M. D., late Demonstrator of Morbid Anatomy at St. Bartholomew's, and a reprint of the English translation of "The Book of the Foundation of St. Bartholomew's." In this reprint the spelling of the original translation is retained. According to this book, "* * * the Priory and the hospital by olde tyme longing to the same," were founded in the year 1119, by one Rayer, in accordance with the command to do so, given him in a vision by the patron saint of the institution. Of the twenty-eight chapters in the book, twenty are devoted to descriptions of the miraculous cures accomplished. In addition to the interest which this reprint has for all St. Bartholomew's men, it is interesting as a sample of English prose of the time of Edward II. The reports cover the general field of medicine and surgery, and represent the experience of the Hospital during the year.

The following is a list of the titles of the articles contained in this volume, viz.: On the Amount of Destructive Impulses in the Insane, Cases Resembling General Paralysis of the Insane, Note on Tuberculous Tumors of the Larynx, On the Presence of the Tubercle Bacillus in Old Specimens of Diseased Lung, Profuse Non-Fatal Pulmonary Hæmoptysis, Five Cases of Functional Nervous Disorder, Cases from Mr. Willet's Wards, Notes of Three Cases of Coal-Gas Poisoning, The After Treatment of Tracheotomy, Two Cases of Parasitic Hæmaturia, Some Cases of Sclerosis of the Spinal Cord, On the Nature and Origin of Rodent Ulcer, Clinical Contributions to Practical Medicine, Two Contributions to Renal Surgery, Variola as Seen in the Casualty Department, A Contribution to the Topographical Anatomy of the Spinal Cord, From the Department for Diseases of the Larynx, Cases of Mental Disturbance after Operations, A Case of Lead Poisoning With Bosses in the Metacarpal Bones, Parametritis and Abscess of the Liver, The Formation of Abnormal Synovial Cysts in Connection with the Joints, On the Breath Sounds in Health and Disease, Cases from Dr. Church's Wards, Notes on the Six Gifts of Theophilus Philanthropos or Robert Poole, and Proceedings of the Abernethian Society for Winter Sessions of 1884-5.

Dr. T. Claye Shaw, in his article on "The Forecast of Destructive Impulses in the Insane," states the following conclusions, viz.: "That the destructive state is one result of a certain condition of brain, and may exist in its greatest intensity without consciousness. That it is especially frequent in those cases of brain disease attended with weak or irregular action of the heart. That epilepsy, the insanity of old age, puerperal insanity, masturbational insanity, and imbecility, are liable to exhibit it. That inasmuch as brain disease is rarely stationary in its extent, and often proceeds insidiously, it is impossible to deny that any insane person may develop the destructive faculty. That persons may be the reverse of a melancholy disposition and yet most suicidal." Dr. Shaw thinks that in most cases the destructive tendency is due to cerebral anæmia, directly caused by irregularity in the action of the heart. In support of his opinion, he says that it exists in many cases of dyspepsia accompanied by irregular action of the heart, and in the cases of masturbators, and that in cases in which the attacks are preceded by depression and melancholy they may be aborted by the use of stimulants. His concluding sentence is, "However great our experience may then be, we are occasionally deceived by the exhibition of dangerous and destructive acts where they were not expected; but if we keep prominently before us the condition of the heart and the vaso-motor system, with especial reference to their causation of a sudden anæmic state of the brain, we have, in my opinion, a trustworthy warning which will justify us in taking steps which we shall find afterwards no reason to regret."

By the term "old specimens of diseased lungs," Dr. Harris refers to specimens preserved in alcohol for many years. He reports examinations of ten specimens taken from the museum, all put up before the year 1846, and some probably as early as the year 1812. The method of staining, which was most successful, was the Ehrlich-Weigert. In one specimen of about the year 1812, no bacilli were found, but in all of the others they were found abundant. Dr. Harris promises a further account of other specimens.

Seven cases of membranous laryngitis, in which the symptoms were sufficiently urgent to necessitate tracheotomy, are reported by S. Herbert Habersham, M.B. Two of these resulted fatally, through conditions entirely beyond control, and independent of the tracheotomy. The cases are reported to illustrate, first, the theory that the treatment of the throat is of much less importance than the nourishment of the patient, and second, to describe the methods used to feed the patients, and to clear the trachea in those cases in which this is necessary. In feeding, during the time when the child will not swallow sufficient food for its nourishment, liquid food was used, and from two to four ounces given every two to four hours, according to the indications in each case. In one case reported, that of a child two and one-half years old, it was observed that just before the feeding time the patient was subject to fits of dyspnœa and coughing, apparently due to exhaustion. Upon the food being given every three, instead of every four hours, these symptoms disappeared, and did not return. The method of introducing the food into stomach is by means of a brass syringe fitted to an ordinary number 4 or 6 English flexible catheter, which is passed through the nose into the stomach. Dr. Habersham says that the introduction of the tube is quite easy. It is indeed difficult to pass it into the trachea, and that when it does it will pass only a few inches, and that the irritation produced is a sure indication for its withdrawal. The introduction is usually easy after the first attempt, and upon one occasion a patient was fed during sleep without being awakened. In introducing the tube the manipulation is exactly that of the introduction of the eustachian catheter. For the purpose of cleaning the trachea, the closed end of a soft-rubber catheter, No. 4 to 6, is clipped off and then connected with a small brass syringe. "It is rapidly passed through the tracheotomy tube down the trachea as far as possible, regardless of the patient's increased dyspnœa, and firmly nipped by the finger and thumb just outside the opening into the trachea. The nurse holding the syringe is then directed to exhaust it by drawing up the handle rapidly. When this is done the finger and thumb are suddenly let go, and the whole force of suction is transferred to the end of the catheter in the trachea. The catheter is then slowly withdrawn." The method as described is very efficient, and may be repeated as frequently as necessary and exhausts the child less than its own ineffectual efforts at expiration.

In this, his second, communication on The Formation of Abnormal Synovial Cysts in Connection with the Joints, Mr. W. Morrant Baker calls attention to his first communication, which appeared in the 13th volume of the St. Bartholemew's Hospital Reports. In all of the cases reported in the first paper the knee joint was involved. In the present communication two cases are reported in which the cysts occurred in connection with the shoulder-joint, two with the elbow-joint, one with the hip-joint, and one over and below the external malleolus. Mr. Baker quotes the conclusions stated in his first report, describes the cases mentioned above, and draws the following conclusions from them:

1. That abnormal synovial cysts may be found in connection, not only with the knee, but in connection with the shoulder, the elbow, the hip, and the ankle-joints.

2. That the manner of the formation of these synovial cysts probably resembles that which has been proved to occur in connection with the knee-joint, namely, that the synovial fluid, upon reaching a certain amount of tension, by accumulation within the joint, finds its way out in the direction of least resistance, either by the channel by which some normal bursa communicates with the joint, or, in the absence of any such channel, by forming first a hernia of the synovial membrane. In both cases, should the tension continue or increase, the fluid at length escapes from the sac, and its boundaries are then formed only by the muscles and other tissues between and amongst which it accumulates.

3. That in the case of the shoulder-joint the abnormal synovial cyst may be found either in front a little below the clavicle, or in the upper arm in the region of the biceps muscle.

4. That in connection with the elbow-joint the cyst is usually placed on the inner side, a little above the internal condyle of the humerus.

5. That in the case of the wrist-joint the synovial cyst may be either in front or behind.

6. In the only case in connection with the hip, in which a note has been preserved, the swelling was in the upper part of Scarpa's triangle.

7. In the one case in connection with the ankle-joint the synovial cyst was in front and to the outer side.

8. That the apparent want of direct communication between the joint and the abnormal synovial cyst is frequently deceptive, and should not lead to the inference that no such communication exists.

9. That the caution given in the previous communication, not to interfere by operation with these synovial sacs without good reason, has been justified by increased experience. "Hitherto I have not discovered any relationship between the form of osteo-arthritis with which some of these synovial cysts are associated, and locomotor ataxy, but I suspect that in some of them a relationship will be found to exist."

The article contributed by Mr. Bullar, "On the Breath Sounds in Health and Disease," is an extension of an account of some similar experiments which appeared in the Proceedings of the Royal Society, No. 234. The article is accompanied by illustrations of the apparatus used, and the details of the experiments made very clearly described. The nature of the investigations is so technical that a condensation of the article cannot fairly represent it. The author claims, and has proved, that the vesicular murmur is produced where the smallest bronchioles open into the vesicles. The experiments further showed that a lung either moderately filled or distended with air, not itself in motion, is a poor conductor of sound, while one moderately or fully collapsed is a fairly good conductor.

The experiment to determine how the vesicular murmur is produced, resulted only in the negative conclusion that it is not by the movement of the tissues of the lung—Mr. Bullar's article is well worthy of the attention of all who have a special interest in the subject discussed.

"Statistical tables of the patients under treatment in the wards of St. Bartholomew's during 1884," finish the volume, and the whole is furnished with a fairly good index.

E. WING.

